# Performance Evaluation of Stone Mastic Asphalt Involving Coarse Steel Slag and Fine RAP

**DOI:** 10.3390/ma18112598

**Published:** 2025-06-02

**Authors:** Yan Wu, Weidong Cao, Chao Xu, Fanshuo Meng, Guangyong Wang, Shutang Liu

**Affiliations:** 1 Qilu Expressway Co., Ltd., Jinan 250100, China; wuyan@qlecl.com (Y.W.); xuchao940616@163.com (C.X.); 2 School of Qilu Transportation, Shandong University, Jinan 250002, China; 202435531@mail.sdu.edu.cn (F.M.); gtreesl@sdu.edu.cn (S.L.); 3 Shandong Transportation Research Institute, Jinan 250102, China; wgy1853@163.com

**Keywords:** stone matrix asphalt (SMA), steel slag, reclaimed asphalt pavement (RAP), mix design, pavement performance

## Abstract

Stone mastic asphalt (SMA) is the most widely adopted asphalt mixture on highway pavement in China. However, the cost of SMA is rising continually due to the increasing shortage of high-quality basalt aggregate. On the other hand, China’s steel slag and reclaimed asphalt pavement (RAP) stock is abundant, and steel slag has excellent strength and wear-resistant performance, which can fully or partially replace part of the basalt aggregate. The content of asphalt may be increased due to the porosity of the steel slag. If fine RAP rich in asphalt is also used for SMA, it can partially fill the voids of steel slag and reduce the amount of new asphalt and fine aggregate. For this objective, SMA 13 was designed with two particle sizes of coarse steel slag aggregate (5–10 mm, 10–15 mm) and one fine RAP (0–5 mm), named SR-SMA. The fundamental pavement performance of SR-SMA was evaluated through a wheel-tracking test, low-temperature beam bending test, freeze–thaw indirect tensile test, and four-point bending fatigue test. For comparison, the mix design and performance tests of two SMAs involving coarse steel slag and fine basalt aggregate (named SB-SMA), and coarse and fine basalt aggregates (named B-SMA), respectively, were conducted. The results indicated that SR-SMA (dynamic stability of 4865 passes/mm) shows the best rutting resistance, followed by SB-SMA (dynamic stability of 4312 passes/mm), and B-SMA (dynamic stability of 4135 passes/mm) comes in last. Additionally, the dynamic stability values of three SMAs have significant differences. SR-SMA has better low-temperature cracking resistance with a failure strain of 3150 με, between SB-SMA and B-SMA (failure strain values are 4436, 2608 με). Compared to B-SMA and SB-SMA, the moisture stability of SR-SMA is relatively poor but meets Chinese specification. While the fatigue resistance of SR-SMA is the worst among three SMAs, their differences are insignificant. Furthermore, SR-SMA reduces material cost by approximately 35% per ton compared to conventional B-SMA. Overall, SR-SMA is cost-effective and can be used as an alternative material to traditional B-SMA.

## 1. Introduction

Stone mastic asphalt (SMA) was developed in Germany in the 1960s and is currently one of the most used asphalt mixes in highway and urban road pavement in China. To achieve a stable coarse aggregate skeleton structure in SMA, superior rock (i.e., basalt) is always needed. However, with the ever-increasing project scale of highway construction and maintenance in China, high-quality rock resources are becoming scarce, leading to the production cost of SMA staying at a high level. On the other hand, China has a large number of steel slag (SS) storages, and steel slag shows better soundness, abrasion resistance, and adhesion to asphalt compared to conventional basalt and limestone aggregates. Therefore, using steel slag as an alternative to natural aggregates in SMA is practical and can bring good environmental and economic benefits [1,2,3,4,5,6,7,8]. However, the abundant surface porous texture of steel slag may result in increased asphalt binder consumption, thus raising the cost of binder in SMA [9]. In China, on the other hand, approximately 220 million tons of reclaimed asphalt pavement (RAP) are produced every year [10]. Additionally, fine RAP usually contains a high content of aged asphalt. If coarse steel slag and fine RAP are applied to SMA simultaneously, the respective advantages of steel slag and fine RAP can be exploited. The partial aged asphalt in RAP may transfer to the steel slag surface and fill some pores when the hot coarse steel slag blends with fine RAP before the addition of virgin asphalt. Therefore, the fresh asphalt content of SMA may be reduced. Meanwhile, fine aggregates of SMA can be completely or partially replaced by the aggregates in fine RAP. The main motivation of this paper is to simultaneously apply both SS and RAP to SMA.

Numerous studies on the separate use of steel slag and RAP in asphalt mixes have been reported [11,12,13], while there have been few extensive studies on the simultaneous incorporation of steel slag and RAP materials into asphalt mixes. Simultaneous incorporation of steel slag and RAP materials into warm mix asphalt (WMA) has been proven to be an economic, environment-friendly, and high-performance option compared to conventional WMA [14,15]. With respect to the control mixture (composed of exclusively natural aggregate), asphalt mixtures with RAP aggregate and Electric Arc Furnace (EAF) steel slag indicate improved fatigue properties and performance [16,17]. Furthermore, steel slag and RAP can be applied to porous asphalt mixtures. Pascale et al. [18] conducted an assessment of the mechanical performance and environmental effect of porous asphalt mixtures with EAF steel slags and RAP aggregates. They concluded that the use of RAP and/or steel slag as a substitute for virgin aggregates (i.e., basalt aggregates) has no effect on the volumetric properties of the porous asphalts, and the combined use of EAF steel slag and RAP decreases the environmental burdens by up to 25% in all impact categories compared to the conventional and reference porous asphalt mixtures. In addition, in terms of mechanical performance, two porous mixtures made with EAF steel slag and RAP showed higher values than the asphalt concrete made with only virgin aggregates. The performance of two kinds of recycled asphalt mixtures (referred to as steel slag RAM and basalt RAM), composed of steel slag and basalt coarse aggregates, respectively, was evaluated in the laboratory by Wang et al. [19]. Steel slag RAM displays a larger comprehensive evaluation function (f) value compared to basalt RAM under the same RAP dosage, indicating its more desirable comprehensive road performance. Shen et al. [20] also conducted research on the performance and mechanical properties of recycled asphalt concrete mixed with steel slag. The results showed that the high-temperature performance of SMA and AC recycled asphalt concrete mixed with steel slag first decreases and then increases with the increase of RAP content, while the low-temperature performance and water stability show a continuous decreasing trend. Additionally, performance and interaction mechanisms of recycled asphalt mixtures involving high RAP content and steel slag were studied by Yang et al. [21,22]. Their research indicates that incorporating steel slag in recycled asphalt mixtures with 70% RAP content shows desirable performance indices in contrast to basalt. The steel slag-recycled asphalt interface, with a higher comprehensive evaluation index of 0.9155, reveals superior comprehensive adhesion performance compared to basalt-recycled asphalt. Furthermore, epoxy asphalt (EA) and steel slag were used together to enhance the performance and increase the RAP content of recycled asphalt mixtures (RAM). In terms of dynamic stability, British Pendulum Number, and texture depth, EA and steel slag significantly enhanced the road performance of steel slag–epoxy-recycled asphalt mixtures [23].

Inspired by previous research and the concept of reducing costs and increasing efficiency, the main objective of this paper was to evaluate the performance of SMA with a 13.2 mm nominal maximum aggregate size, composed of 100% SS coarse aggregate and 100% RAP fine aggregate (named SR-SMA). For comparative analysis, SMAs made with 100% steel slag coarse aggregate and 100% fine basalt aggregate (named SB-SMA) and 100% basalt aggregates (named B-SMA) were also tested. Wheel-tracking tests, beam bending tests, freeze–thaw indirect tensile tests, and four-point bending fatigue tests were performed in the laboratory to characterize high-temperature rutting resistance, low-temperature cracking resistance, water stability, and fatigue resistance. In addition, a simple cost analysis was conducted for the three SMA mixtures.

## 2. Materials and Mix Design

### 2.1. Raw Materials

#### 2.1.1. Steel Slag

**Figure 1:**
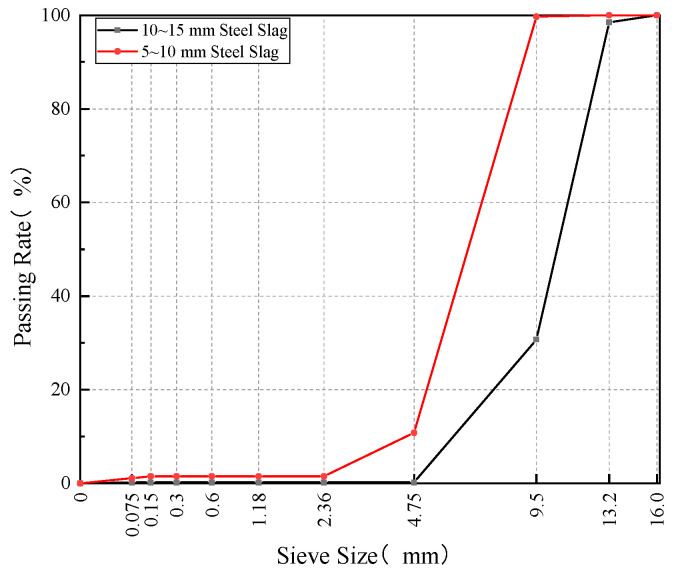
Sieving results of two particle sizes of steel slag.

In this study, steel slag was utilized as the coarse aggregate. Two size ranges of steel slag coarse aggregate (5–10 mm and 10–15 mm) were selected. The basic properties and sieving results of the two steel slag particle sizes are shown in Table 1 and Figure 1, respectively.

Generally, steel slag has potential volume instability, primarily caused by expansion due to the hydration of free calcium oxide (f-CaO) and free magnesium oxide (f-MgO), along with the corresponding products of Ca(OH)_2_ and Mg(OH)_2_. This reaction may lead to cracking when steel slag is incorporated into asphalt mixtures. Therefore, the volumetric stability of steel slag was assessed before use. Water-immersion expansion testing and f-CaO content determination were used as assessment methods. The tests were conducted in accordance with the Test Method for Stability of Steel Slag (GB/T 24175-2009) [24] and the Determination of Free Calcium Oxide Content in Steel Slag (YB/T 4328-2012) [25].

The average water-immersion expansion rate is 0.9%, and the average free CaO content is 0.7%. Both values meet the requirements (i.e., water-immersion expansion rate ≤ 1.8%, and free CaO content ≤ 3%) specified in Steel Slag for Asphalt Mixture (JT/T 1086-2016) [26].

#### 2.1.2. RAP

**Figure 2:**
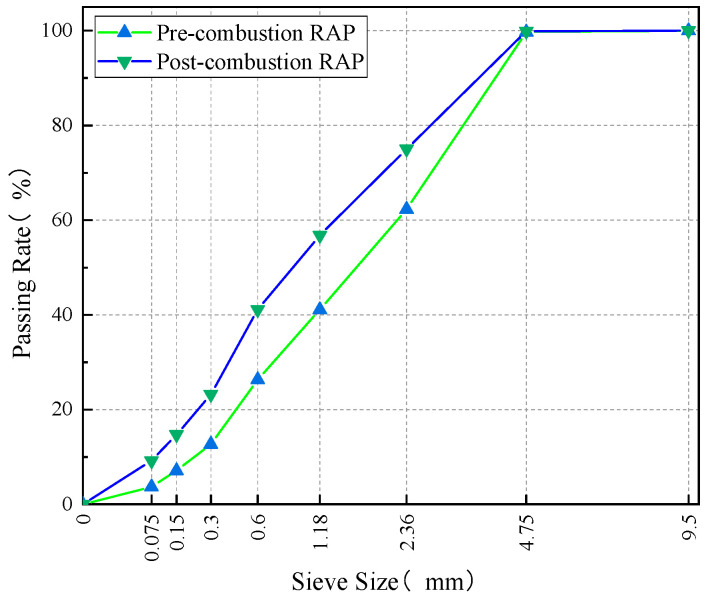
Sieving curves before and after RAP combustion.

RAP supplied by a maintenance company in Shandong Province was used in this study. The crushed fines portion (0–5 mm) of RAP was selected for testing. To evaluate the performance of the aged asphalt in the RAP, the asphalt was extracted and recovered in accordance with China Standard Test Methods [27]. The basic performance indicators of the aged asphalt were then tested. The test results are presented in Table 2. It can be seen from Table 2 that the degree of aging of aged asphalt in RAP is not severe, and it can be used for hot central plant recycling [28]. The asphalt content in RAP was determined using the combustion furnace method according to China Standard Test Methods JTG E20-2011, Section T 0735-2011 [27], and the burned RAP aggregate was subjected to sieve analysis. The average aged asphalt content is 6.8%. The apparent specific gravity and bulk specific gravity of the RAP aggregate are 2.746 and 2.603, respectively. Aggregate sieving results before and after RAP combustion are shown in Figure 2.

#### 2.1.3. Other Materials

SBS-modified asphalt was used in the laboratory, and its basic performance indicators were tested in accordance with China Standard Test Methods [27]. The test results are presented in Table 3. All performance indicators meet the requirements specified in Chinese specifications [29]. Basalt was used as the new aggregate in this study, including two sizes of coarse aggregate (10–15 mm and 5–10 mm) and one size of fine aggregate (0–5 mm). All aggregates were sourced from asphalt mixing plants located in the vicinity of Jinan, Shandong Province. Limestone mineral powder was used as the filler material. The basic physical and mechanical properties of the basalt aggregates and the mineral powder are presented in Table 4 and Table 5, respectively.

A fiber is commonly incorporated in SMA mixtures to prevent drain-down of the asphalt binder during construction. In this study, lignin fibers were used, which were obtained from a local supplier in Jinan, Shandong Province. Their performance indicators were tested, and the results are presented in Table 6.

### 2.2. Mix Design

#### 2.2.1. Design of SR-SMA Mix Proportion

**Figure 3:**
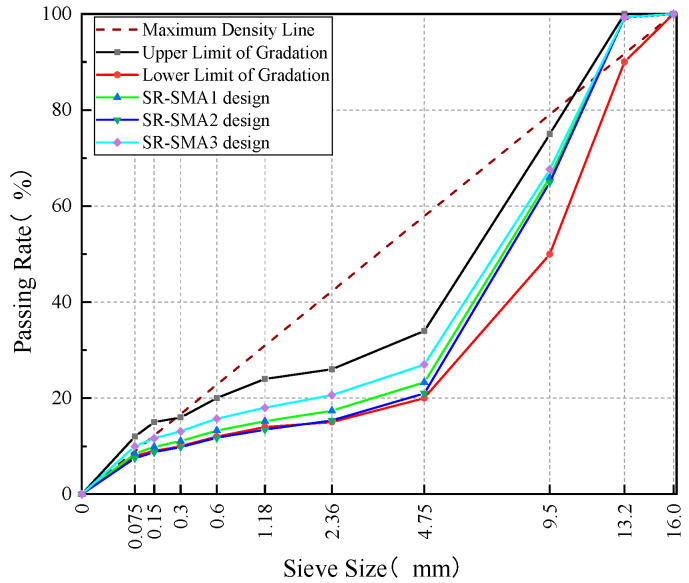
SR-SMA gradation curves.

In accordance with the Technical Specification for Highway Asphalt Pavement Construction [29], three initial gradations (SR-SMA1, SR-SMA2, and SR-SMA3)—corresponding to 4.75 mm sieve passing rates of approximately 21%, 24%, and 27%, respectively—were proposed, as shown in Figure 3. The contents of RAP were 10.6%~14.4% by weight of total mineral aggregates for three initial gradations. The initial asphalt-to-aggregate ratio was determined to be 6.13% based on the estimation formula and engineering experience [30]. The contribution of the aged RAP binder is 0.72%~0.98% in the total asphalt-to-aggregate ratio. Since the RAP asphalt binder is not aged severely and its content in SR-SMA is very small, the blending chart and rejuvenating agent were not used in this study.

According to China Specifications [28], the preparation process of Marshall specimens was as follows: First, the RAP was heated to 120 °C in a drying oven, and the heating time was controlled within 2 h. Meanwhile, the new aggregates (i.e., steel slag) were heated to 190~195 °C in a drying oven. Then, the heated RAP and steel slag were put into the mixing pot together and blended for about 1 min. Subsequently, the heated SBS-modified asphalt (about 180 °C) was poured into the mixing pot and continuously blended for about 1 min. Last, the heated mineral powder was added and blended until the mixture was thoroughly mixed. The total blending time of the above mixture was about 3 min. Then, three groups of specimens were manufactured and key volumetric parameters such as volume of air voids (VV), voids in mineral aggregate (VMA), and voids filled with asphalt (VFA) were measured according to the Marshall test method [27]. The results of volumetric parameters of compacted specimens for SR-SMA are shown in Table 7. In Table 7, γ_sa_ is synthetic apparent relative gravity of mineral aggregates; γ_t_ is theoretical maximum relative gravity of asphalt mixture; and γ_f_ is bulk relative gravity of compacted asphalt mixture.

From Table 7, SR-SMA1 was found to exhibit a VV of 3.81%, which was closest to the design target of 4% [29]. Additionally, the values of VMA and VFA for SR-SMA1 all met the specification requirements (i.e., VMA ≥ 16.5% and VFA of 75%~85%) [29]. Thus, SR-SMA1 was selected as the design gradation. The asphalt-to-aggregate ratio including new and old asphalt binders was determined to be 6.1%.

#### 2.2.2. Design of SB-SMA Mix Proportion

**Figure 4:**
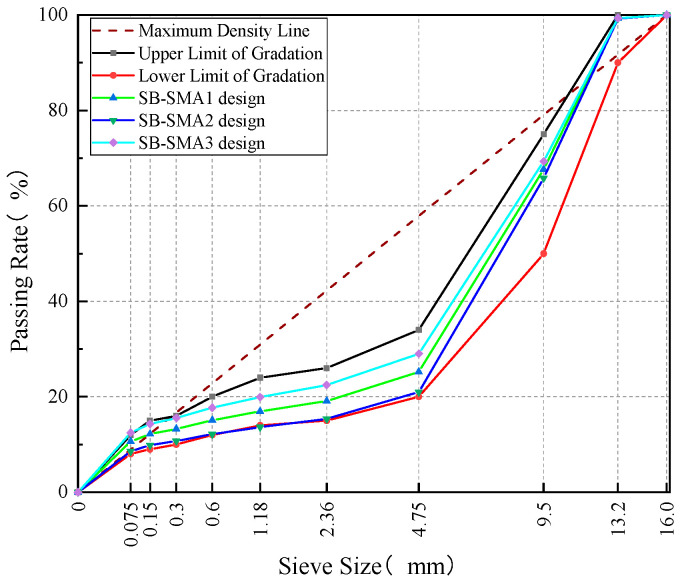
SB-SMA gradation curves.

Steel slag was used as the coarse aggregate in the SB-SMA mixture, while basalt was adopted as the fine aggregate and limestone powder was employed as mineral filler. Similarly, according to Technical Specification for Construction of Highway Asphalt Pavement [29], three gradations (4.75 mm passing rate of about 21%, 25%, 29%) were initially proposed, shown in Figure 4. The initial asphalt-to-aggregate ratio was determined to be 5.8% by using the formula and referring to experience. Likewise, three groups of specimens were prepared, and key volumetric parameters were determined by the Marshall test method, and the results are shown in Table 8.

Based on the analysis and comparison of volume parameters in Table 8, SB-SMA1 was identified as the design gradation. The corresponding optimum asphalt-to-aggregate ratio was determined to be 5.9% according to the procedure in China Specification [29].

#### 2.2.3. Design of B-SMA Mix Proportion

**Figure 5:**
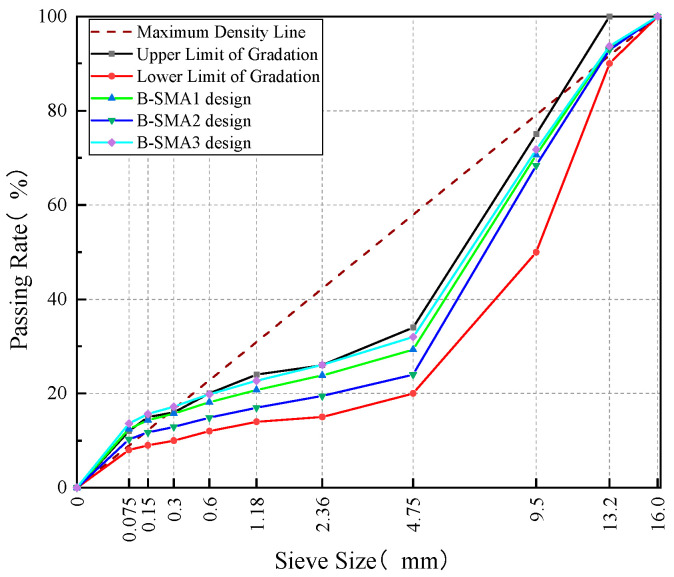
B-SMA gradation curves.

In the B-SMA mixture, both coarse and fine aggregates were composed of basalt, and limestone powder was used as the mineral filler. The procedure of optimization design for B-SMA mix proportion was the same as that for SR-SMA and SB-SMA, so the detailed process is not repeated here. Three initial gradations were proposed in Figure 5, and the results of volumetric parameters of compacted specimens for B-SMA with an initial asphalt-to-aggregate ratio of 6.3% are shown in Table 9. B-SMA1 was selected as the optimal gradation, and the optimum asphalt-to-aggregate ratio was determined to be 6.3%.

## 3. Test Program and Methods

### 3.1. Scanning Electronic Microscope Test

To analyze the microscopic morphology of steel slag and basalt aggregate, a scanning electron microscope electronic microscope (SEM) test was conducted to characterize the surface microstructure of the aggregate using an FE-SEM (Zeiss Sigma 300, manufacturer: Carl Zeiss AG, Oberkochen, Germany).

### 3.2. Performance Tests

In order to compare and analyze the road performance of the three SMAs, a wheel-tracking test, beam bending test, freeze–thaw indirect tensile test, and four-point bending fatigue test were carried out in the laboratory. The high-temperature, anti-rutting ability of asphalt mixture was evaluated by the wheel-tracking test. The testing indicator is dynamic stability (DS), calculated by Equation (1) as follows [27]:

(1) $$DS=\frac{\left(t_{2}-t_{1}\right)\times N}{d_{2}-d_{1}}$$

where $d_{1}$ is rut depth at $t_{1}$ (45 min), mm; $d_{2}$ is rut depth at $t_{2}$ (60 min), mm; N is speed of wheel passing over the center of the sample, 42 cycles a minute.

In general, the higher the DS, the stronger the ability to resist high-temperature deformation; thus, the better the high-temperature stability. The test was conducted in accordance with China Standard Test Methods [27].

The low-temperature beam bending test was used to evaluate the low-temperature crack resistance of the asphalt mixture, and the test indicator was the maximum bending strain (i.e., failure strain). The failure strain can be calculated using Equation (2).

(2) $$\varepsilon_{B}=\frac{6hd}{L^{2}}$$

where $\varepsilon_{B}$ is failure strain, με; h is height of section at midspan, mm; d is displacement at midspan, mm; and L is span of specimen, mm.

The failure strain can characterize the ability of asphalt mixture to resist bending deformation at low temperatures. The larger the value, the stronger the ability of asphalt mixture to resist cracking deformation at low temperatures. The test temperature was −10 °C ± 0.5 °C and the loading rate was 50 mm/min. The test was performed per China Standard Test Methods JTG E20-2011, Section T 0715-2011 [27].

The freeze–thaw indirect tensile test was used to evaluate the water stability of the asphalt mixture. The test index was indirect tensile strength (ITS) before and after freeze–thaw, and the evaluation index was tensile strength ratio (TSR). The indirect tensile strength ratio (TSR) was calculated by the ratio of the original strength that was retained after the moisture conditioning using Equation (3). The test process was carried out according to China Standard Test Methods JTG E20-2011, Section T 0729-2000 [27].

(3) $$TSR=\frac{ITS_{c}}{ITS_{d}}$$

where $ITS_{c}$ is the average value of indirect tensile strength of conditioned group, MPa, and $ITS_{d}$ is the average value of indirect tensile strength of dry group, MPa.

The four-point bending fatigue test of asphalt mixture is used to evaluate the fatigue resistance of the asphalt mixture. The test temperature is 15 °C ± 0.5 °C, loading frequency is 10 Hz, and the continuous sinusoidal loading mode controlled by constant strain is adopted. The test process is carried out according to China Standard Test Methods JTG E20-2011, Section T 0739-2011 [27].

## 4. Results and Discussion

### 4.1. Microscopic Analysis of Aggregate

**Figure 6:**
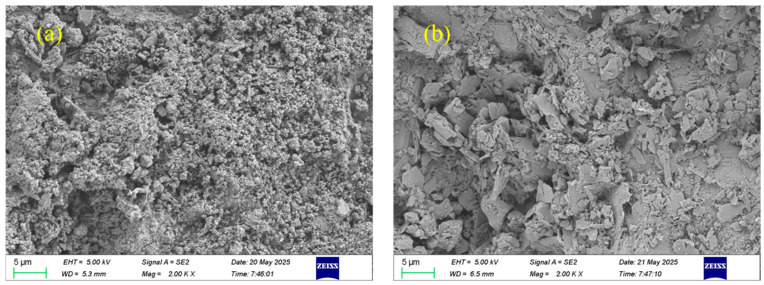
SEM images of aggregates: (**a**) steel slag, (**b**) basalt.

The surface microstructure images of steel slag and basalt aggregates are shown in Figure 6. From Figure 6a, it can be clearly seen that the surface of steel slag exhibits a distinct porous and agglomerated morphology, characterized by heterogeneous particle size distribution and loosely packed particles, resulting in notably high surface roughness. This highly irregular and porous microstructure significantly enhances the mechanical interlocking between the steel slag aggregate and asphalt binder, while also facilitating asphalt infiltration into the surface texture. Consequently, interfacial adhesion is effectively reinforced. Additionally, the increased specific surface area of steel slag offers abundant active sites for adhesion, further promoting asphalt wetting and encapsulation.

In contrast, as shown in Figure 6b, basalt aggregate predominantly displays plate-like or block-shaped regular geometries with sharp edges and relatively smooth, flat surfaces, lacking prominent pore structures. Although such morphology contributes to the formation of a stable aggregate skeleton, the limited surface roughness weakens the potential for mechanical interlocking with asphalt, thereby resulting in relatively lower interfacial bonding strength.

### 4.2. High-Temperature Rutting Resistance Performance

**Figure 7:**
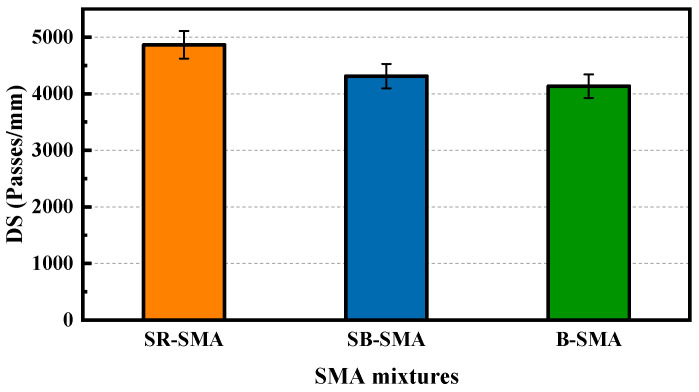
Results of wheel-tracking test for three SMAs.

Three specimens for each SMA in the wheel-tracking test were carried out, and the results are shown in Figure 7 (the error bars shown in the Figure represent standard deviations).

As shown in Figure 7, the dynamic stability values of the three SMAs meet the requirements of the Specification [29]. The dynamic stability value (dynamic stability of 4865 passes/mm) of SR-SMA is the highest and its high-temperature performance is the best. Compared with the dynamic stability of B-SMA (dynamic stability of 4135 passes/mm), SR-SMA increased by 17.6%, and SB-SMA (dynamic stability of 4312 passes/mm) increased by 4.3%. The optimal high-temperature performance of SR-SMA attributed to the mechanical strength of steel slag is higher than that of basalt and contains some aged asphalt binder. Additionally, the above microscopic analysis of aggregate showed the interfacial adhesion between steel slag and asphalt binder is better than that of basalt aggregate, resulting in excellent rutting resistance for SR-SMA.

The analysis of variance (ANOVA) was performed to determine the effect of the type of SMA on dynamic stability (i.e., rutting resistance). The level of significance is 0.05 (α = 0.05) in the ANOVA, with the results shown in Table 10. *F* is greater than that of *F*_critical_ in Table 10, indicating that the type of SMA has a significant effect on DS.

### 4.3. Low-Temperature Cracking Resistance Performance

**Figure 8:**
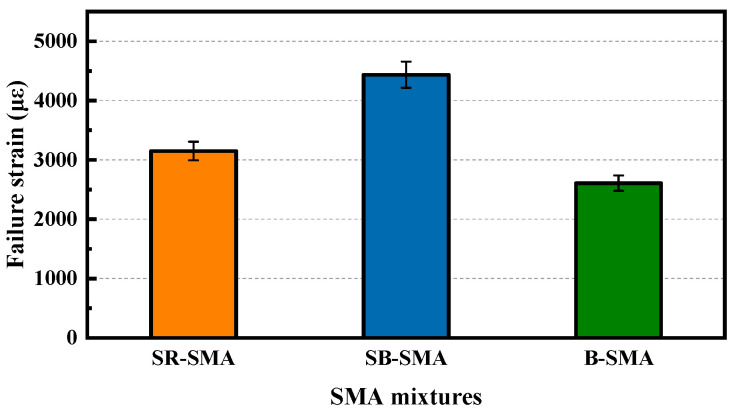
Results of low-temperature beam bending test for three SMAs.

The results of the low-temperature beam bending test of the three SMAs (4 specimens for each mixture) are shown in Figure 8.

Based on the failure strain value in Figure 8, the low-temperature performance of the three SMA mixtures is ranked as BS-SMA, SR-SMA, and B-SMA. However, all of them meet the low-temperature performance requirements for modified asphalt mixtures in winter cold areas specified in China Specification [29]. Compared with the B-SMA, the failure strain value of SB-SMA is 1.7 times that of B-SMA, and the failure strain value of SR-SMA is 1.21 times that of B-SMA. This may be attributed to the improved adhesion between steel slag and asphalt. Although SR-SMA contains some fine RAP, its small content has minimal effect on low-temperature performance. Therefore, the low-temperature performance of SR-SMA is better than that of B-SMA. However, due to the influence of the aged asphalt in RAP, its low-temperature performance is lower than that of SB-SMA.

The ANOVA was also conducted to determine the effect of the type of SMA on failure strain (i.e., low-temperature cracking resistance). Table 11 presents the ANOVA results for failure strain. From Table 11, it can be seen that the type of SMA has a significant effect on failure strain.

### 4.4. Water Stability

**Figure 9:**
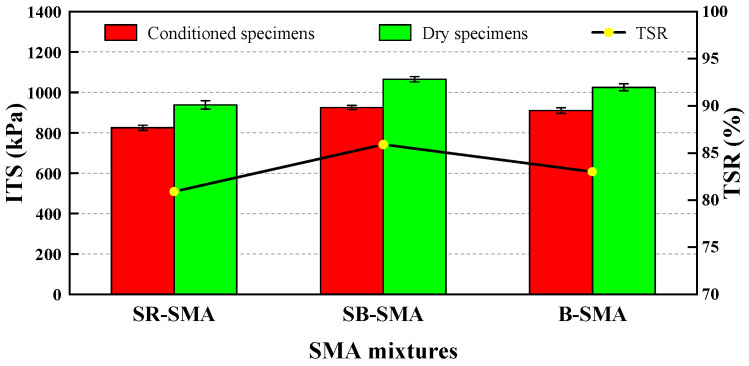
Results of indirect tensile strength test for three SMAs.

The indirect tensile strength test of four specimens, respectively, was carried out before and after the freeze–thaw cycle for each SMA, and the average values of indirect tensile strength of the dry group and conditioned group were taken to determine the TSR. The results are shown in Figure 9.

It can be seen from Figure 9 that the TSR values of all SMAs meet the requirements of China Specification [29]. Among them, the TSR of SB-SMA is the highest, indicating the best water stability, while the SR-SMA exhibits the lowest, and B-SMA is between them. This may be due to SR-SMA containing about 12% RAP, and the adhesion of asphalt binder (containing aged asphalt) to RAP fine aggregate decreasing under freeze–thaw conditions. Therefore, based on the water stability consideration, the addition of RAP should be limited to a maximum allowable amount. Table 12 shows the ANOVA results of the indirect tensile strength for three SMAs. It is found that the type of SMA has a significant effect on the moisture susceptibility at the 5% level.

### 4.5. Fatigue Resistance Performance

**Figure 10:**
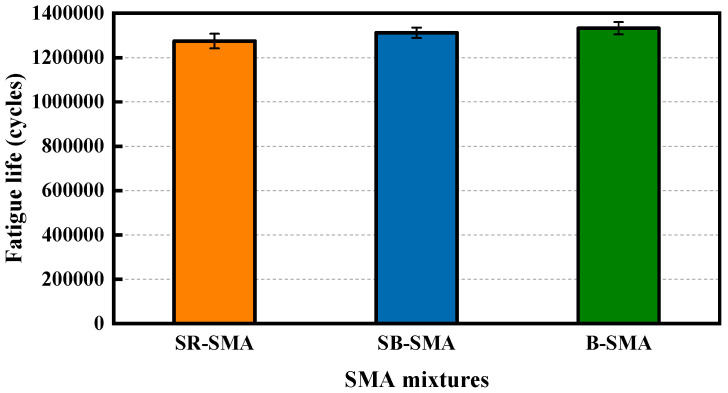
Fatigue life of three SMAs.

Due to time constraints, the four-point bending fatigue test was performed only at a strain of 400 με. The fatigue life of the three SMAs, represented by the number of loading cycles (the average of four specimens), is shown in Figure 10. It can be seen from Figure 10 that SR-SMA exhibits the shortest fatigue life among the three SMAs. This may be attributed to the simultaneous application of steel slag and RAP in SR-SMA, resulting in the greatest initial stiffness modulus for SR-SMA. At the same strain level, the SR-SMA experiences the highest stress, leading to the fastest decrease in modulus, and consequently the least cumulative loading cycles.

Similarly, the ANOVA for fatigue life of various SMAs was also conducted, and the results are shown in Table 13. It is observed from Table 13 that fatigue life is statistically insignificant within each SMA mixture. In general, the SMA type is found to have no significant effect on fatigue resistance performance at the 5% level. Admittedly, the fatigue resistance performance of SR-SMA needs to be further tested and verified at various strain levels.

## 5. Economic Cost Comparison

Taking the determined aggregate gradations of SR-SMA and B-SMA as an example, the local stone unit price is applied to analyze and compare the project’s economic benefits from the perspective of material costs only. The SR-SMA asphalt-to-aggregate ratio is 6.0%, and the fiber content is 0.3%. The asphalt-to-aggregate ratio of B-SMA is 6.3%, with a fiber content of 0.3%. Assuming that the steel slag and basalt material yard is 50 km from the asphalt mixing station, the asphalt mixing station is 10 km from the construction site, and the freight rate is unified at 1 yuan per kilometer per ton, the cost per ton of the two types of SMAs is calculated based on their respective proportions and material unit prices, as shown in Table 14. The material unit prices of steel slag, basalt, RAP, mineral powder, fiber, and modified asphalt are estimated based on those in Shandong Province for that year.

It can be seen from Table 14 that the unit cost of SR-SMA is 35% lower than that of B-SMA. Therefore, SR-SMA significantly reduces the project cost while also contributing to resource conservation and environmental protection, offering substantial economic and social benefits.

## 6. Conclusions

The main objective of this research was to evaluate the performance of stone mastic asphalt composed of 100% steel slag coarse aggregate and 100% RAP fine aggregate (SR-SMA). For comparative analysis, two other SMAs, made with 100% steel slag coarse aggregate and 100% fine basalt aggregate (SB-SMA), and total basalt aggregates (B-SMA) were also conducted. The fundamental performance of three SMAs was evaluated through a wheel-tracking test, beam bending test, freeze–thaw indirect tensile test, and four-point bending fatigue test. The main conclusions are summarized as follows:

1. SR-SMA demonstrates the highest dynamic stability of 4865 passes/mm, followed by SB-SMA with a dynamic stability of 4312 passes/mm and B-SMA with a dynamic stability of 4135 passes/mm, indicating superior high-temperature rutting resistance of SR-SMA. Furthermore, the type of SMA has a significant effect on dynamic stability.
2. In terms of low-temperature cracking resistance evaluated by beam bending test, the ranking is SB-SMA, SR-SMA, and B-SMA. SR-SMA shows better cracking resistance than the conventional B-SMA, although slightly lower than SB-SMA due to the presence of aged asphalt in RAP. Furthermore, the SMA type is found to have a significant effect on failure strain.
3. Although SR-SMA shows relatively lower water stability than B-SMA and SB-SMA, it meets specification requirements. In terms of fatigue life at a strain of 400 με, the fatigue resistance of the three mixtures is ranked as B-SMA, SB-SMA, and SR-SMA. However, fatigue life is statistically insignificant within each SMA mixture.
4. Compared to conventional B-SMA, SR-SMA reduces material cost by approximately 35% per ton of mixture. Overall, SR-SMA is a cost-effective and sustainable alternative to conventional B-SMA.

The present study primarily focused on a comprehensive evaluation of the fundamental pavement performance of SR-SMA with one kind of RAP and steel slag. It should be noted that the findings and conclusions in the paper need to be further verified using various RAP and steel slags. Additionally, SR-SMA’s environmental benefits can be evaluated through a life cycle assessment in future research.

## Figures and Tables

**Table 1:** Basic properties of steel slag.

Steel Slag (mm)	Apparent Specific Gravity	Bulk Specific Gravity	Water Absorption (%)	Crushing Value (%)	Percent of Flat and Elongated Particles (%)
5–10	3.613	3.425	1.52	—	5.6
10–15	3.569	3.452	0.95	11.6	6.0

**Table 2:** Test results of aged asphalt properties.

Test Items	Unit	Test Results	Specification Requirements [28]
Penetration (25 °C, 5 s, 100 g)	0.1 mm	38.8	≥10
Ductility (15 °C, 5 cm/min)	cm	10.2	—
Softening point	°C	56.5	—

**Table 3:** Technical performance indices of SBS-modified asphalt.

Test Indicators	Unit	Test Results	Technical Requirements [29]	Test Method
Penetration (25 °C, 100 g, 5 s)	0.1mm	58	40–60	JTG E20-2011, Section T0604-2011
Softening point	°C	80.2	≥55	JTG E20-2011, Section T0606-2011
Ductility (5 cm/min, 5 °C)	cm	30.7	≥30	JTG E20-2011, Section T0605-2011
Brookfield viscosity (135 °C)	Pa·s	2.5	<3	JTG E20-2011, Section T0625-2011
Storage stability segregation, 48 h softening point difference	°C	2.0	≤2.5	JTG E20-2011, Section T0661-2011

**Table 4:** Test results of technical indicators of basalt aggregate.

Technical Indicators	Particle Size/mm		
	10~15	5~10	0~5
Bulk specific gravity	2.797	2.753	2.575
Apparent specific gravity	2.892	2.894	2.879
Water absorption (%)	1.175	1.778	2.543
Crushing value (%)	13.6	—	—
Needle flake particle content (%)	4.4	6.2	—

**Table 5:** Technical indicators of mineral powder.

Items		Unit	Test Results	Technical Requirements [29]
Water content		%	0.3	≤1.0
Apparent specific gravity		—	2.821	≥2.50
Appearance		—	No agglomerates	No agglomerates
Heating stability		—	Qualified	Qualified
Particle size range	<0.6 mm	%	100	100
	<0.15 mm	%	91.4	90~100
	<0.075 mm	%	79.6	75~100

**Table 6:** Lignin fiber performance test results.

Items	Unit	Test Results	Technical Requirements [29]
Fiber length	mm	4.6	≤6
Ash content	%	17.2	18 ± 5
pH value	—	7.3	7.5 ± 1.0
Oil absorption rate	—	Qualified (5.4 times)	≥5 times of fiber mass
Moisture content	%	3.1	≤5

**Table 7:** SR-SMA volume parameters.

Gradation Curves	γ_sa_	γ_t_	γ_f_	VV (%)	VMA (%)	VFA (%)
SR-SMA1	3.390	2.914	2.803	3.81	17.14	83.92
SR-SMA2	3.412	2.931	2.810	4.28	17.45	75.60
SR-SMA3	3.354	2.878	2.857	0.72	14.94	95.23

**Table 8:** SB-SMA volume parameters.

Gradation Curves	γ_sa_	γ_t_	γ_f_	VV (%)	VMA (%)	VFA (%)
SB-SMA1	3.398	2.960	2.840	4.05	16.61	75.60
SB-SMA2	3.435	2.981	2.834	4.93	17.40	71.73
SB-SMA3	3.374	2.941	2.786	5.29	17.67	70.10

**Table 9:** Volume parameters of B-SMA.

Gradation Curves	γ_sa_	γ_t_	γ_f_	VV (%)	VMA (%)	VFA (%)
B-SMA1	2.885	2.552	2.452	3.92	17.07	77.06
B-SMA2	2.857	2.530	2.413	4.64	17.22	73.06
B-SMA3	2.885	2.550	2.459	3.55	16.36	78.30

**Table 10:** Results of ANOVA of DS.

	SS	df	MS	*F*	*F*_critical_	*p*-Value
Source of variation (DS)						
Between	870,038.0	2	435,019.0	8.798216	5.143253	0.016440
Within	296,664.0	6	49,444.0			
Total	1,166,702.0	8				

**Table 11:** Results of ANOVA of failure strain (α = 0.05).

	SS	df	MS	*F*	*F*_critical_	*p*-Value
Source of variation						
Between	7,057,013.8	2	3,528,506.9	93.607844	4.256495	0.000001
Within	339,251.1	9	37,694.6			
Total	7,396,264.9	11				

**Table 12:** Results of ANOVA of ITS (α = 0.05).

Samples	*F* Value	*F*_critical_ Value	Significance
Conditioned specimens	57.224299	4.256495	Yes
Dry specimens	74.785714		Yes

**Table 13:** Results of ANOVA of fatigue life (α = 0.05).

	SS	df	MS	*F*	*F*_critical_	*p*-Value	Significance
Source of variation							No
Between	5,146,313,058.7	2	2,573,156,529.3	3.160971	5.143253	0.115456	
Within	4,884,239,887.3	6	814,039,981.2				
Total	10,030,552,946.0	8					

**Table 14:** Comparison of the unit cost for two SMAs.

Materials	Steel Slag	Basalt	RAP	Mineral Powder	Fiber	Modified Asphalt	SR-SMA	B-SMA
Unit cost (yuan/ton)	50	170	60	85	3000	4930	311.92	481.40

## Data Availability

The original contributions presented in the study are included in the article, further inquiries can be directed to the corresponding author.
